# Primary Neuroendocrine Tumor of the Left Hepatic Duct: A Case Report with Review of the Literature

**DOI:** 10.1155/2012/786432

**Published:** 2012-11-18

**Authors:** Ajay H. Bhandarwar, Taher A. Shaikh, Ashok D. Borisa, Jaydeep H. Palep, Arun S. Patil, Aditya A. Manke

**Affiliations:** Division of GI and HPP Surgery, Department of Surgery, Grant Medical College & Sir JJ Group of Hospitals, Byculla, Mumbai 400008, India

## Abstract

Primary Biliary Tract Neuroendocrine tumors (NET) are extremely rare tumors with only 77 cases been reported in the literature till now. We describe a case of a left hepatic duct NET and review the literature for this rare malignancy. To the best of our knowledge the present case is the first reported case of a left hepatic duct NET in the literature. In spite of availability of advanced diagnostic tools like Computerized Tomography (CT) Scan and Endoscopic Retrograde Cholangio Pancreaticography (ERCP) a definitive diagnosis of these tumors is possible only after an accurate histopathologic diagnosis of operative specimens with immunohistochemistry and electron microscopy. Though surgical excision remains the gold standard treatment for such tumors, patients with unresectable tumors have good survival with newer biologic agents like Octreotride.

## 1. Introduction

NET is derived from the embryonal neural crest cells called Argentaffin or Kulchitsky cells and have a potential for secreting serotonin. This tumor can arise anywhere in the distribution of the Argentaffin cell system. In addition to the most common sites of occurrence, namely, ileum and appendix these tumors have reported to occur in bladder, prostate, rectum, stomach, bronchi, pancreas, and biliary tree.

Primary Biliary Tract NETs are rare and account for 0.2%–2% [1, 2] of all gastrointestinal neuroendocrine tumors.

The Literature documents about 77 cases of neuroendocrine tumor arising from the biliary tree which includes common bile duct, common hepatic duct, cystic duct, and hilar confluence.

We report a case of Primary Biliary NET arising from the left hepatic duct. An extensive search of the literature has yielded no reference regarding a NET arising from the left Hepatic ducts. The present case is the first reported case of a NET arising in the left hepatic duct.

## 2. Case Report

**Figure 1:**
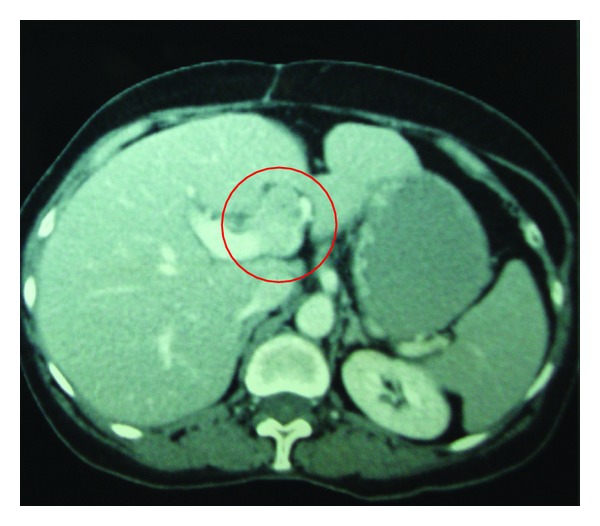
CT scan showing mass lesion in segment 4 abutting the left branch of portal vein.

**Figure 2:**
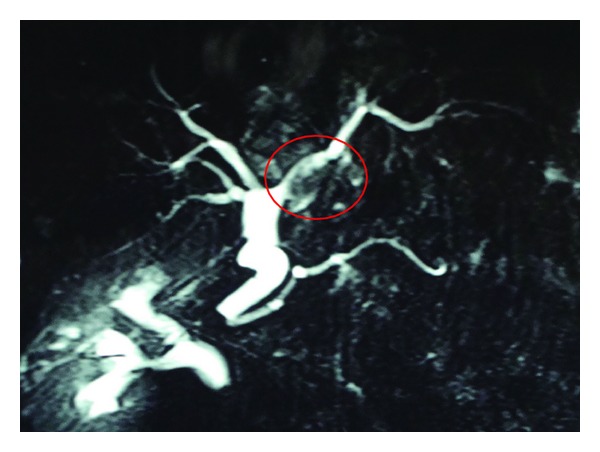
MRCP showing a filling defect in the left hepatic duct.

**Figure 3:**
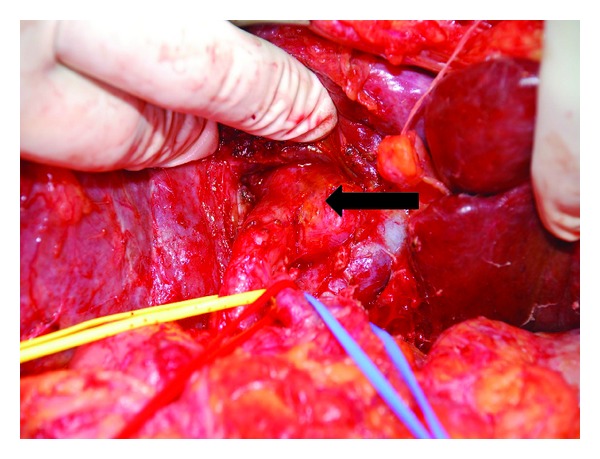
Intraoperative picture showing tumor arising from the left hepatic duct. CBD hooked in yellow, hepatic artery in red, and portal vein in blue.

**Figure 4:**
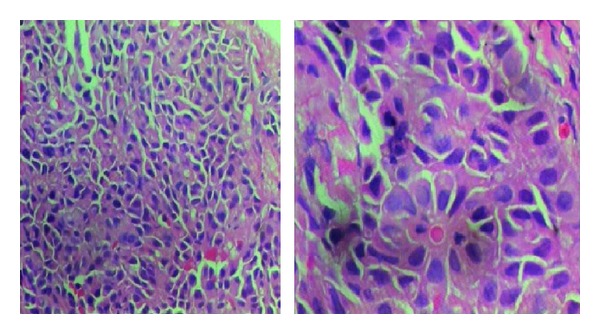
Histopathology showing a typical rosette appearance of neuroendocrine tumor.

**Figure 5:**
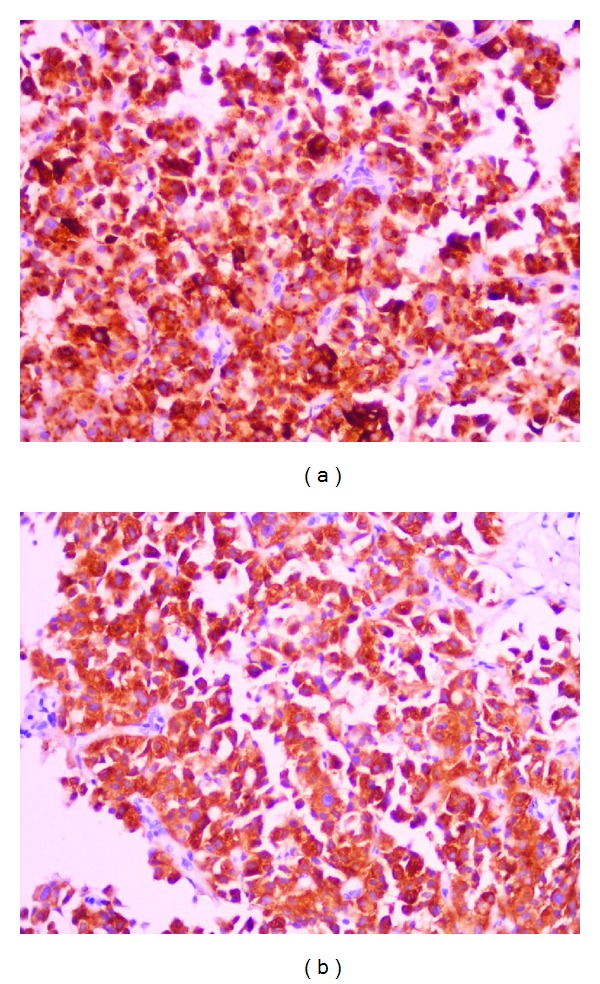
Immunohistochemistry staining positive for Chromogranin (a) and Synaptophysin (b).

**Figure 6:**
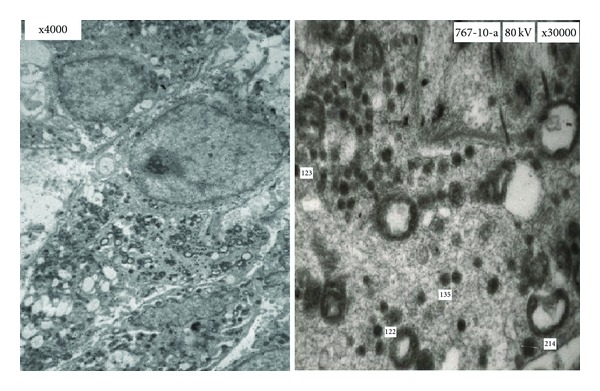
Electron microscopy picture showing multiple granules of varying sizes.

A 69-years-old female presented with colicky pain in the right hypochondrium since 3 years. She had past history of open cholecystectomy done for gall stones 15 years back. On physical examination the patient was anicteric with soft abdomen. An Ultrasonography (USG) of the abdomen showed a hypoechoic lesion of size 3.5 cm × 4 cm in segment 4 of the liver. Computerized Tomography (CT) of the abdomen showed a 4.1 × 3.7 cm heterogeneously enhancing mass lesion in segment IV of liver abutting the left branch of portal vein (Figure 1). A Magnetic Resonance Imaging (MRI) of upper abdomen with Magnetic Resonance CholagioPancreaticography (MRCP) showed a filling defect in the left hepatic duct with lesion in the adjacent part of liver in segment IV, suggestive of a left hepatic duct tumor with infiltration in the liver (Figure 2). Tumor marker serum alfa fetoprotein was mildly raised. Patient was worked up for left hepatectomy. Intraopertaively a lesion arising from the left hepatic duct involving the left branch of portal vein and extending upto the portal confluence was found (Figure 3) rendering the tumor unresectable. A biopsy was taken from the mass and procedure was abandoned in view of inoperability. Histopathology showed typical rosette appearance of a neuroendocrine tumor (Figure 4) and immunohistochemistry positive for CD56, Chromogranin and Synaptophysin (Figure 5). Ultrastructural study of the cell with electron microscopy (Figure 6) showed the presence of multiple neurosecretory granules with muscle tissue.

**Figure 7:**
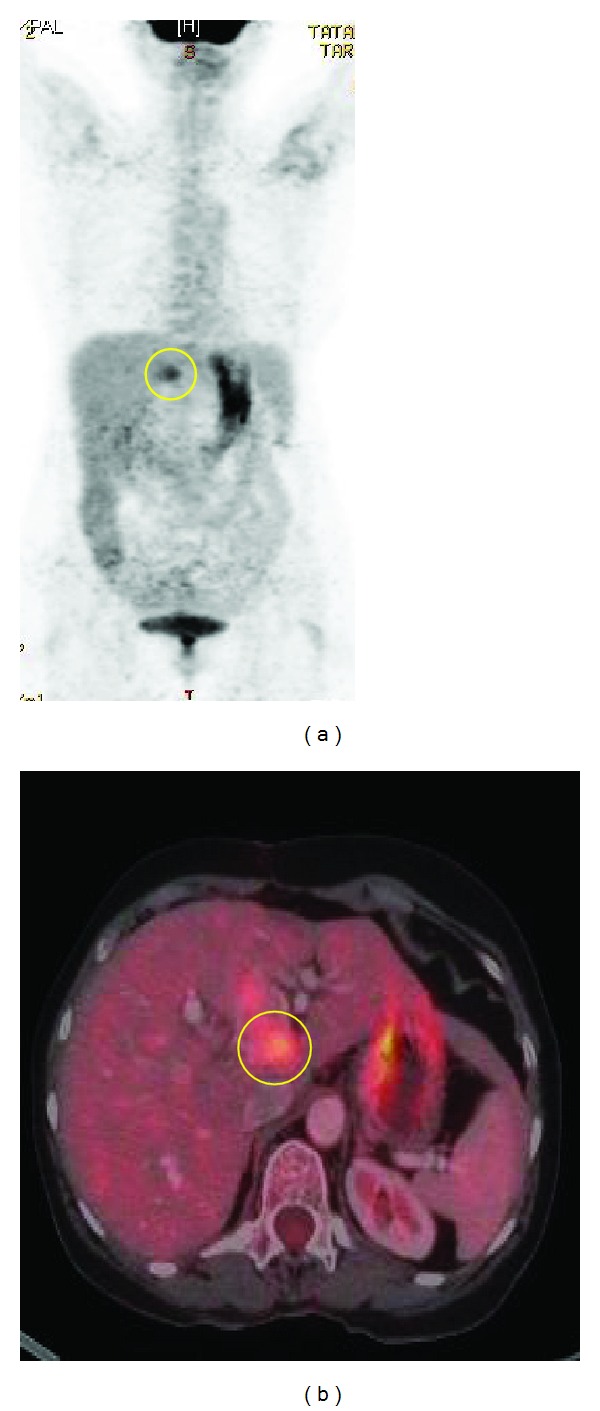
PET Scan (a) and Octreotride labelled (b) scan showing tumor limited to the hepatobiliary system.

A whole body Positron Emission Tomography (PET) scan and an Octreotride labeled radionulceotide scan showed somatostatin receptor expressing lesion in the hepatobiliary system (Figure 7). 

**Figure 8:**
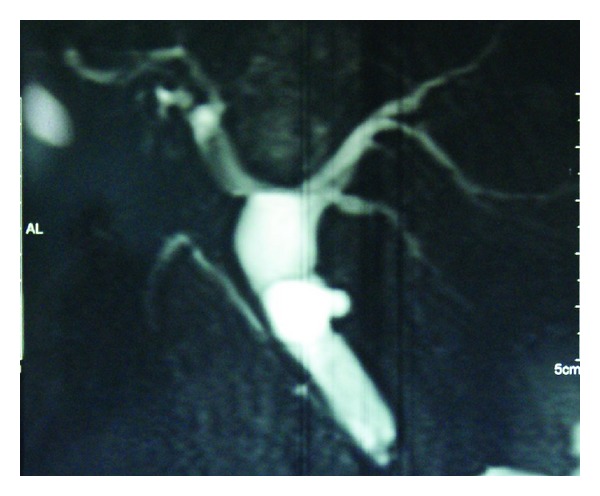
Follow up MRCP at 1 year showing absence of any filling defect in the left hepatic duct.

The patient was started on long-acting Octreotride therapy single dose every month. The patient has received 12 of such doses and is doing well after a 1-year followup without any complications. A follow up MRI (Figure 8) upper abdomen with MRCP at 1 year showed the absence of filling defect in the Left hepatic duct that was seen previously which showed tumor regression.

## 3. Discussion

Primary Biliary-tract NETs are very rare. They account for 0.2–2% of all gastrointestinal NET [1, 2] reason being the paucity of enterochromaffin cells from which NETs arise in this area. Chronic inflammation of the bile duct epithelium is responsible for metaplasia of these enterochromaffin cells and formation of NET.

Davies [3] in 1959 reported NET of the distal bile duct and pancreatic duct which represented more of a periampullary NET rather than a biliary tract. Pilz [4] in 1961 was credited to report the first case of a Biliary Tract NET. After an extensive search of the Medline only 77 cases of Biliary Tract NET have been reported so far in the literature since 1961 (Table 1).

Till now no NET in the literature has been reported in the isolated left hepatic duct possibly making our case the first reported case of an isolated left hepatic duct NET.

The most common site of malignancy in the biliary tract was common bile duct (57.14%) followed by the hilar confluence (27.28%), the cystic duct (9.1%), common hepatic duct (5.12%) and finally the left hepatic duct (1.23%). 

The mean age of presentation was 47 years (range 6 years to 79 years).

The male to female ratio is 1 : 1.23 showing that the biliary NET has a preponderance for female.

By far the most common symptom in patients of Biliary tract NET is Jaundice (63.4%) followed by Pain (14.1%), jaundice with pain (12.7%) and remaining nonspecific symptoms like weight loss.

The incidence of a Carcinoid syndrome in patients of Biliary Tract NET is very rare. Only 4 cases which include a single case published by Nesi et al. [51] in 2006 with symptoms of diarrhea due to secretion of serotonin and 3 cases by Price et al. [65] in 2009 with features of Zollinger Ellison syndrome due to secretion of gastrin from tumor in CBD.

## 4. Conclusion

Biliary Tract NET are rare tumors that typically present with jaundice and pain. As compared to its counterpart Cholangiocarcinoma Biliary NET occurs in a younger age group with a female preponderance [39]. Biliary NET usually are nonsecreting tumor. Preoperative diagnosis of these tumors require a high index of suspicion and accurate histopathological diagnosis which must include a immunohistochemistry study and electron microscopy. Biliary tract NET are slow-growing indolent tumor which have a limited propensity for local and metastatic spread. Surgical resection aimed at complete tumor excision with bilio-enteric continuity offers the best cure and high survival rates. Patients who have undergone resection have a long term survival. Even in inoperable patients chemotherapy with newer biologic agents like Octreotride have a favorable outcome on the patient's survival.

## Figures and Tables

**Table 1:** Showing study of reported cases of Biliary tract NET.

No.	Case reference	Age	Sex	Complaint	Location
(1)	Pilz, [4] 1961	55	F	Weakness	CBD
(2)	Little et al., [5] 1968	41	F	RUQ pain, jaundice,	Hilar
(3)	Bergdahl, [6] 1976	79	F	Autopsy finding	Distal CBD
(4)	Judge et al., [7] 1976	19	M	Jaundice, pain	Hilar
(5)	Gerlock and Muhletaler [8] 1979	32	M	Jaundice	CBD
(6)	Vitaux et al., [9] 1981	24	M	Jaundice	Distal CBD
(7)	Nakamuara et al. [10] 1981	58	F	Jaundice	CBD
(8)	Abe et al., [11] 1983	64	M	RUQ pain	CBD
(9)	Goodman et al., [12] 1984	28	F	RUQ Pain	Cystic duct
(10)	Jutte et al., [13] 1986	62	M	Back Pain	CHD
(11)	Nicolescu and Popescu, [14] 1986	50	F	RUQ pain	CBD
(12)	Alexander et al., [15] 1986	64	F	Hematemesis	CBD
(13)	Gastinger et al. [16] 1987	65	F	Jaundice, Pain	Hilar
(14)	Reinhardt et al. [17] 1988	71	F	Jaundice, fever	CBD
(15)	Chittal and Ra, [18] 1989	46	F	RUQ pain	Cystic duct
(16)	Fujita et al., [19] 1989	55	F	RUQ pain	CBD
(17)	Bickerstaff and Ross [20] 1989	57	F	Jaundice	CBD
(18)	Brown et al., [21] 1990	35	F	Jaundice	Hilar
(19)	Bumin et al., [22] 1990	38	F	Jaundice	CBD
(20)	Fellows et al. [23] 1990	30	M	Jaundice	CBD
(21)	Besznyák et al. [24]	13	F	Jaundice	Hilar
(22)	Angeles-Angeles et al., [25] 1991	39	F	Jaundice	CBD
(23)	Barron-Rodriguez et al., [26] 1991	36	M	Jaundice, RUQ pain	CBD
(24)	Newman et al., 1992 [27]	15	F	N/A	CBD
(25)	Dixon et al., 1992 [28]	60	F	RUQ pain	CBD
(26)	Rugge et al., [29] 1992	64	F	Jaundice, RUQ pain	Cysticduct, CBD
(27)	Gembala et al., [30] 1993	28	M	Jaundice	Hilar
(28)	Mandujano-Vera et al., [31] 1995	53	F	Jaundice	CBD
(29)	Sankary et al., [32] 1995	47	F	Jaundice	Hilar
(30)	Hao et al., [33] 1996	47	M	Incidental finding	CBD
(31)	Kopelman et al., [34] 1996	44	M	Jaundice	CBD
(32)	Belli et al., [35] 1996 CBD	78	M	Jaundice	CBD
(33)	Bembenek et al., [36] 1998	12	F	Jaundice	Hilar
(34)	Nahas et al., [37] 1998	61	F	Jaundice	Hilar
(35)	Ross et al., [38] 1999	65	F	Jaundice	CBD
(36)	Chamberlain and Blumgart [39] 1999	37	F	Itching	Hilar
(37)	Chamberlain and Blumgart [39] 1999	67	F	Itching	Hilar
(38)	Hermina et al., [40] 1999	69	M	RUQ pain	Cystic duct
(39)	Perakath et al. [42] 1999	36	F	Jaundice, Pain	CHD
(40)	Chan et al [41] 2000	14	M	Jaundice	Hilar
(41)	Maitra et al., [42] 2000	53	F	Jaundice,	CBD
(42)	Maitra et al., [42] 2000	61	F	Jaundice, itching	Hilar
(43)	Juturi et al., [43] 2000	43	M	Jaundice, itching	CBD
(44)	Turrión et al., [44] 2002	51	F	Jaundice, itching	Hilar
(45)	Pawlik et al., [45] 2003	59	M	Jaundice	Hilar
(46)	Podnos et al., [46] 2003	65	F	Cholecystitis	CBD
(47)	Podnos et al., [46] 2003	27	M	Jaundice, itching	CBD
(48)	Volpe et al., [47] 2003	19	M	Jaundice, pain	CBD
(49)	Menezes et al., [48]	30	M	Jaundice	CHD
(50)	Ligato et al., [49] 2005	33	F	Irritable bowel	Hilar
(51)	Hubert et al., [50] 2005	NA	M	Jaundice	CBD
(52)	Hubert et al., [50] 2005	NA	M	Jaundice	CBD
(53)	Hubert et al., [50] 2005	NA	F	Jaundice	CBD
(54)	Nesi et al., [51] 2006	30	M	Jaundice, iarrhoea	CBD
(55)	Kim et al., [52] 2006	67	F	Jaundice	CBD
(56)	Caglikulekci et al., [53] 2006	40	F	Jaundice	Hilar
(57)	Honda et al., [54] 2006	76	M	Jaundice, pain	CBD
(58)	Todorki et al., [55] 2007	73	M	Jaundice, Fever	CBD
(59)	Sethi et al., [56] 2007	51	M	ERCP finding	CHD with Cystic
(60)	Stavridi et al., [57] 2007	NA	NA	NA	Cystic duct
(61)	Jiménez et al., [58] 2007	60	M	Jaundice	Hilar
(62)	Ferrone et al., [59] 2007	NA	NA	NA	NA
(63)	Nafidi et al., [60] 2008	31	F	RUQ pain	CBD
(64)	Gusani et al., [61] 2008	NA	NA	NA	CBD
(65)	Schmitt et al., [62] 2008	NA	NA	NA	Hilar
(66)	Costantini et al., [63] 2008	NA	NA	NA	CHD
(67)	Felekouras et al., [64] 2009	60	F	Jaundice	Cystic duct
(68)	Price et al., [65] 2009	NA	NA	Jaundice	CBD
(69)	Price et al., [65] 2009	NA	NA	Jaundice	CBD
(70)	Price et al., [65] 2009	NA	NA	Jaundice	Hilar
(71)	Tonnhofer et al., [66]	6	F	Jaundice	CBD
(72)	Zhan et al. [67] 2010	10	M	NA	CBD
(73)	Squillaci et al., [68] 2010	52	M	Jaundice	CBD
(74)	Squillaci et al., [68] 2010	70	M	Jaundice	CBD
(75)	Tsalis et al., [69] 2010	77	M	Incidental	Hilar
(76)	Lee et al., [70] 2011	59	M	Jaundice	CBD
(77)	Athanasopoulos et al., [71] 2011	43	M	Jaundice	CBD
(78)	Present case	69	F	Pain	Left hepatic duct

CHD: common hepatic duct, CBD: common bile duct, Hilar at the common bile duct bifurcation.
